# Barcoding Beetles: A Regional Survey of 1872 Species Reveals High Identification Success and Unusually Deep Interspecific Divergences

**DOI:** 10.1371/journal.pone.0108651

**Published:** 2014-09-25

**Authors:** Mikko Pentinsaari, Paul D. N. Hebert, Marko Mutanen

**Affiliations:** 1 Department of Biology, University of Oulu, Oulu, Finland; 2 Biodiversity Institute of Ontario, University of Guelph, Guelph, Ontario, Canada; 3 Department of Biology, University of Oulu, Oulu, Finland; Consiglio Nazionale delle Ricerche (CNR), Italy

## Abstract

With 400 K described species, beetles (Insecta: Coleoptera) represent the most diverse order in the animal kingdom. Although the study of their diversity currently represents a major challenge, DNA barcodes may provide a functional, standardized tool for their identification. To evaluate this possibility, we performed the first comprehensive test of the effectiveness of DNA barcodes as a tool for beetle identification by sequencing the COI barcode region from 1872 North European species. We examined intraspecific divergences, identification success and the effects of sample size on variation observed within and between species. A high proportion (98.3%) of these species possessed distinctive barcode sequence arrays. Moreover, the sequence divergences between nearest neighbor species were considerably higher than those reported for the only other insect order, Lepidoptera, which has seen intensive analysis (11.99% vs up to 5.80% mean NN divergence). Although maximum intraspecific divergence increased and average divergence between nearest neighbors decreased with increasing sampling effort, these trends rarely hampered identification by DNA barcodes due to deep sequence divergences between most species. The Barcode Index Number system in BOLD coincided strongly with known species boundaries with perfect matches between species and BINs in 92.1% of all cases. In addition, DNA barcode analysis revealed the likely occurrence of about 20 overlooked species. The current results indicate that DNA barcodes distinguish species of beetles remarkably well, establishing their potential to provide an effective identification tool for this order and to accelerate the discovery of new beetle species.

## Introduction

With almost 400,000 described species [1], beetles (Coleoptera) are the most speciose order of animals. Estimates of undescribed beetle diversity vary considerably ([2], [3]), but it is agreed that many species await description. For example, about 62,000 species of the relatively well-studied weevils (Curculionoidea) have been described, but the total species count is estimated to be about 220,000 [4]. On this basis, it is likely that total number of beetle species exceeds one million. As might be expected from their high diversity, beetles show an extraordinary variety of life histories, habitat and diet and occur in all biogeographical regions except for mainland Antarctica. Their ecological importance is immense, and many are important pests of cultivated plants, stored products and timber ([5], [6], [7], [8]). Some beetle species serve as important model organisms, including *Tribolium castaneum* (Herbst, 1797) [9] and *Callosobruchus maculatus* (Fabricius, 1775) [10], [11].

The vast number of animal, fungal, plant and protist species creates an insurmountable barrier for any comprehensive effort to catalogue biodiversity by means of traditional taxonomy. Triggered by this realization, often called the taxonomic impediment ([12], [13], [14]), Tautz et al. in 2003 [12] proposed an entirely DNA-based system for the delineation of species. Almost simultaneously, Hebert *et al.* [15] suggested DNA barcoding as a universal DNA-based identification system for species that was designed to reinforce the capacity of Linnean taxonomy. Since the original proposal, DNA barcoding has become a global enterprise with more than 3000 publications on the theme. Large-scale projects targeting specific geographical regions or taxonomic groups have been established, such as Fauna Bavarica (http://www.faunabavarica.de/), NorBOL (http://www.norbol.org/), GBOL (https://www.bolgermany.de/), the Lepidoptera Barcode of Life (http://www.lepbarcoding.org/) and FISH-BOL (http://www.fishbol.org/), initiatives which are contributing to the International Barcode of Life Project (http://ibol.org/). However, despite the momentum that DNA barcoding has gained, beetles have received little attention. Some studies have tackled a particular species group or members of a family within a particular region ([16], [17], [18], [19]), but no taxonomically comprehensive test of the effectiveness of DNA barcoding has been carried out at any geographical scale.

DNA barcoding relies on the assumption that sequences in the 5′ region of the cytochrome *c* oxidase I (COI) gene are more similar among members of a species than to sequences of any other species. For the most part, this seems to be the case ([20], [21], [22]), although increasing the geographical scale of sampling often reduces its success ([23], [24], [25], but see [26]). This decline is due to the increase in number of closely related species and to a rise in genetic variation within species as geographical scale expands and more specimens are analyzed. Sampling can also impact results on a local scale. The proportion of a clade’s local species sampled and the number of specimens sampled per species both contribute to observed variation within and between species. The rate of success of COI barcoding varies across animal lineages. Some cnidarians are, for example, problematic because of their slowed rate of mitochondrial evolution [27] and within insects, there are differences among orders [28].

This paper presents the first taxonomically comprehensive test of the effectiveness of DNA barcodes in beetles using the North European coleopteran fauna as a test group. Because this fauna has seen intensive taxonomic study since Linnaeus, the basic requirement for a strong taxonomic platform to test barcode performance on Coleoptera is undoubtedly better met in North Europe than most other regions on the planet. The fauna comprises about 5400 species based on the most recent checklist for the Nordic and Baltic countries [29]. We also examine how sampling affects the nature of the barcode gap on a regional scale. Finally, we assess the ability of the Barcode Index Number system implemented in BOLD to discriminate the beetle species recognized through the past 250 years of traditional taxonomic work.

## Materials and Methods

### Sampling and barcode sequencing

Most of the specimens examined in this study were freshly collected from the field specifically for DNA barcoding in 2011 and 2012. The specimens were preserved in 70% ethanol as soon as possible after collecting and stored at –20°C until tissue sampling. All sampling was made in accordance with the laws of the countries where the samples were collected. A sampling permit to all government-owned protected areas in Finland was issued to the Finnish Expert Group on Coleoptera including MP by Metsähallitus (Finnish Forest and Park Service, permit number 2322/662/2012). The Centre for Economic Development, Transport and the Environment in Lapland permitted sampling of *Pytho kolwensis* Sahlberg, 1833, a species protected by law in the European Union (permit number LAPELY/275/07.01/2012). Sampling non-protected Coleoptera species outside national parks and other protected areas does not require special permits in the Nordic countries.

In addition to this fresh material, we sampled older pinned specimens from private collections and from the collection of the Zoological Museum at the University of Oulu, Finland. Our sampling efforts form a part of the Finnish Barcode of Life project (FinBOL, http://finbol.org/) which aims to assemble DNA barcodes for all species of animals, plants and fungi occurring in Finland. The present data represent progress toward a comprehensive barcode library for Finnish Coleoptera after two years of work.

DNA was extracted from 6423 specimens representing about 2070 species. Most of these specimens (5855; 91%) were collected from Finland. The others originate from Sweden (317; 5%), Estonia (235; 4%), Russia (15; <1%), and some single specimens from other countries. The sampling localities are situated between 56°N–71°N and 15°E–32°E.

Tissue samples were placed in 96-well microplates and sent to the Canadian Centre for DNA Barcoding (CCDB) for DNA extraction, PCR and sequencing of the 648 bp COI barcode region. Depending on the size and state (fresh/dry) of the sampled individual, one to three whole leg(s), part of a leg, a piece of the thoracic flight muscles or the whole beetle was used for extraction. CCDB’s standard high-throughput protocols (documentation available at http://ccdb.ca/resources.php) were used for extraction, PCR and sequencing. A cocktail of the Folmer primers LCO1490 (GGTCAACAAATCATAAAGATATTGG) and HCO2198 (TAAACTTCAGGGTGACCAAAAAATCA) [30], and the LepF1 (ATTCAACCAATCATAAAGATATTGG) and LepR1 (TAAACTTCTGGATGTCCAAAAAATCA) primers [31] was used in the first PCR amplification attempt for most specimens. If resources allowed, specimens that failed to produce full-length barcode sequences were failure tracked to recover shorter 307 bp and 407 bp sequences using different PCR primer sets. Details of PCR and sequencing primers for all samples, the barcode sequences and the trace files for these sequences were uploaded to the Barcode of Life Data Systems (BOLD) database [32] for storage and analysis along with all relevant collection data and photographs of the specimens.

All sampled specimens were identified to species based on morphology by MP. Whenever a discrepancy between species identification and BIN assignment or sequence placement in a neighbor-joining tree was detected, the identification of the specimen involved was checked to correct misidentifications. In all subsequent analyses we only used those barcode records with a sequence length of at least 500 bp and that also fulfilled the other requirements for barcode compliance [33].

### Sequence statistics and identification success

We used the analysis tools in BOLD to calculate the nucleotide composition of the sequences and distributions of Kimura-2-Parameter distances within and between species. The performance of barcode sequences in species identification was assessed by conducting a barcode gap analysis in BOLD. All species found to share haplotypes with one or more other species were interpreted as identification failures, but the Discussion considers the possibility that some of these cases may reflect oversplitting of species and hence inaccurate current taxonomy.

### Barcode Index Numbers

The Barcode Index Number (BIN) system was created as an interim taxonomic system to aid management of the 3 M barcode sequences in BOLD, especially those records that derive from specimens lacking a species-level scientific name [33]. Sequences are assigned to BINs using the Refined Single Linkage (RESL) algorithm which performs an initial single linkage analysis employing 2.2% sequence divergence as a minimum distance between clusters. The resulting operational taxonomic unit (OTU) boundaries are then refined by Markov clustering [33]. The BIN assignments on BOLD are constantly updated as new sequences are added, and individual BINs can be split or merged in light of new data [33]. The BIN assignments used in this study were downloaded from BOLD on January 24, 2014. We used the comparison scheme presented by Ratnasingham & Hebert [33] to examine the correspondence between traditionally recognized species and the OTUs delimited by the RESL algorithm. Each species was assigned to one of four categories as follows: (1) Match: all specimens of a species included in one BIN; (2) Split: specimens of a single species divided into two or more BINs; (3) Merge: all specimens of two or more species combined into a single BIN; (4) Mixture: Both a merge and a split involving two or more species.

### Sampling effects

The relationship between the number of individuals sampled per species and its maximum sequence divergence was examined by fitting a locally weighted polynomial regression curve (LOESS) with 95% confidence intervals to a scatterplot of these two variables. We also computed Spearman’s rank correlation coefficient for these two variables and tested for significance. One extreme outlier (the jewel beetle *Agrilus viridis* (Linnaeus, 1758), with 44 individuals sampled) was excluded from this analysis because it has been shown to comprise several species [34]. Singletons were also excluded, leaving a total of 1308 species for this analysis.

To test how the completeness of species-level sampling affected the barcoding gap, we made a simple resampling study on the Carabidae, the most comprehensively sampled large family in our dataset (199 of 295 species recorded from Finland represented by one or more sequences). Random sets of 20, 40, 60 … 160 and 180 species were subjected to a barcode gap analysis in BOLD and the average K2P distances to the nearest hetero-specific individuals, as well as the lowest NN divergence observed among the analyzed species, were recorded. MUSCLE [35] was used to align the sequences for each analysis. The sampling and analysis was repeated 10 times for each of the categories (20, 40 … 180), so 90 barcode gap analyses were conducted. The probability of including closely related species pairs or groups in a barcode gap analysis increases as the number of species sampled increases. Accordingly, the minimum nearest neighbor distance observed in a barcode gap analysis should rapidly decrease and the average NN distance should steadily decline, eventually stabilizing as the number of sampled species and the number of included close relatives increases. A LOESS line was fit to the resampling data, and the significance of a Spearman’s correlation coefficient was evaluated to determine the association between the two variables.

Statistical analyses were performed in the R statistical environment v. 3.0.2 using the packages distributed with the basic Windows binaries, as well as the package ggplot2 [36] for producing the graphics and LOESS fitting.

### Availability of material

All barcode sequences and all relevant collection data (localities, coordinates, dates etc.) are available in BOLD (http://www.boldsystems.org), along with voucher deposition details, original sequence trace files and photographs of the specimens. All these data are combined into a single citable dataset (doi: dx.doi.org/10.5883/DS-FBCOL). The sequences are also available in GenBank (see Supporting information, Table S1 for accession numbers).

## Results

Barcode compliant sequences were recovered from 5290 of the 6423 specimens, representing 1872 named species. Shorter, <500 bp sequences were recovered from another 408 specimens. Although these short sequences are not analyzed here, they can still be useful for species identification so they are released for public use along with all the barcode compliant sequences. 694 specimens (10.8% of all specimens) produced no sequence information even after failure tracking, and 39 were found to be contaminated by DNA from other species. Most failures involved museum specimens, some more than ten years old and/or collected with pitfall and flight-interception traps where conditions for DNA preservation are far from optimal. Among fresh specimens, the failure rate was notably high in Dryopidae (48%) and Histeridae (36%), indicating possible PCR primer binding problems in these families.

**Figure 1:**
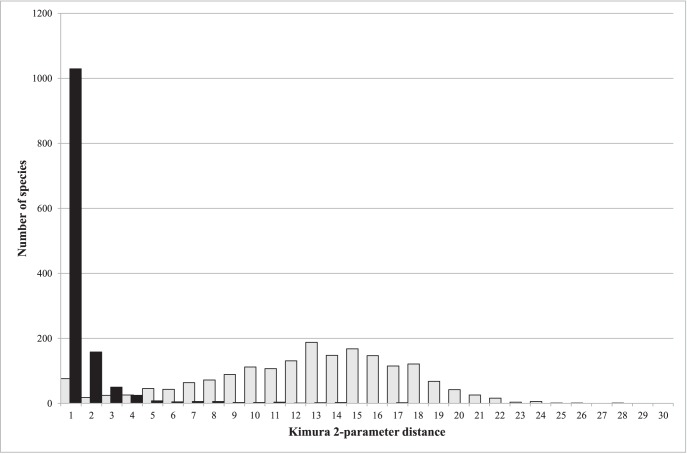
Distributions of Kimura 2-parameter distances within (black) and between species (white).

The 5290 sequences were strongly AT biased, with an overall average AT content of 66.0% (range 55.2–73.7%, Table 1). The bias was especially strong at the third codon position (mean AT content 85.5%, range 60.8–98.2%). The overall mean intraspecific K2P distance was 0.54% (range 0–16.72%), while the mean distance to the nearest neighbor species was 11.99% (range 0–27.61%). When intraspecific values above 10% were excluded (because they likely represent overlooked species), the mean divergence dropped to 0.38%. Fig. 1 shows the distribution of maximum K2P distances within species as well as the distance to the nearest neighbor species. The divergences within and between species for each family are presented in a supplementary table (Table S2).

**Table 1:** Nucleotide composition of the >500 bp sequences used in all the analyses.

	min	mean	max	SE
G %	10.33	16.32	20.52	0.01
C %	12.92	17.66	26.29	0.03
A %	22.19	29.58	35.41	0.02
T %	26.44	36.42	42.86	0.03
GC %	26.29	33.98	44.83	0.04
GC % codon pos 1	31.91	44.86	54.59	0.04
GC % codon pos 2	35.11	42.5	45.89	0.01
GC % codon pos 3	1.82	14.51	39.21	0.08

Thirty one of the 1872 species (1.6%) shared COI haplotypes with at least one other species, preventing their discrimination. Twenty eight of these cases involved 14 pairs of congeners, while the final case involved haplotype sharing by three close related species (Dytiscidae: *Agabus congener* (Thunberg, 1794), *A. lapponicus* (Thomson, 1867) and *A. thomsoni* (J. Sahlberg, 1871)). As a result, 1841 of 1872, or 98.3%, of the species possessed unique haplotypes, allowing their identification. Most of these species (1780) showed more than 2% divergence from their nearest neighbor, but 61 species showed less divergence. Further sampling efforts may reveal more cases of sequence sharing because 52.8% of the species were represented by only 1–2 specimens, and 48.7% by only a single barcode haplotype (Table 2).

**Table 2:** Sampled individuals and observed haplotypes per species.

	individuals			haplotypes		
number of ind./hapl.	species count	%	cumul. %	species count	%	cumul. %
1	562	30.02	30.02	911	48.66	48.66
2	427	22.81	52.83	510	27.24	75.9
3	331	17.68	70.51	239	12.77	88.67
4	219	11.7	82.21	140	7.48	96.15
5	171	9.13	91.34	49	2.62	98.77
6	98	5.24	96.58	14	0.75	99.52
7	35	1.87	98.45	6	0.32	99.84
8	15	0.8	99.25	2	0.11	99.95
9	2	0.11	99.36	0	0	99.95
10	2	0.11	99.47	0	0	99.95
>10	10	0.53	100	1	0.05	100

**Figure 2:**
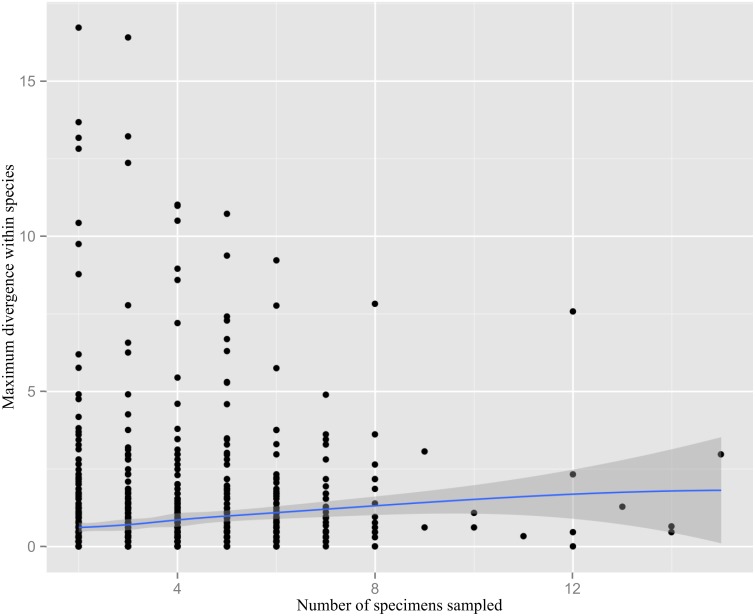
Sample size versus maximum sequence divergence observed within species. The shaded area represents the 95% confidence interval for the fitted LOESS curve.

**Figure 3:**
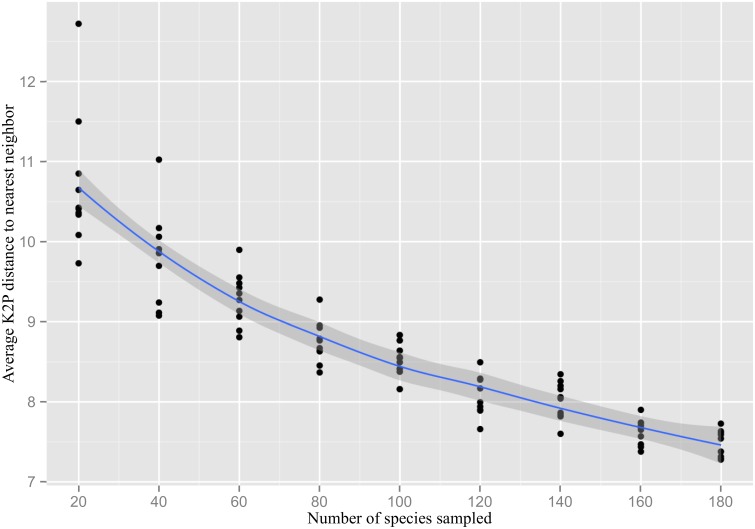
Number of species of Carabidae sampled versus the average sequence divergence (K2P) between nearest neighbor species. The shaded area represents the 95% confidence interval for the fitted LOESS curve.

There was a significant positive correlation between the number of specimens sampled for a species and its maximum intraspecific divergence (Spearman’s rho 0.304, p<0.001). As there are rather few data points with a sample size larger than 8, the confidence interval for the fitted LOESS curve widens towards the right (Fig. 2). The observed average distance between nearest neighbors in the barcode gap analysis for the Carabidae dropped from about 11% to 7.5% with increasing species coverage (Fig. 3). The correlation was highly significant (Spearman’s rho –0.906, p<0.001). The minimum observed distance between species varied considerably in sample sizes below 80 species, but ultimately drops to zero because of the rare cases of no divergence such as *Agonum ericeti* (Panzer, 1809) vs. *A. sexpunctatum* (Linnaeus, 1758) (not shown). The observed average K2P distance to nearest neighbor taxon in the complete set of 199 carabid species was 7.28%.

The correspondence between traditionally recognized species and BINs delimited by the RESL algorithm was strong. In fact, 1724 of 1872 species (92.1%) fall in the match category, with all specimens of a single species and no specimens of other species in that BIN. Cases of conflict involved 107 (5.7%), 38 (2.0%) and 3 (0.2%) species that were assigned to the split, merge and mixture categories, respectively. Close inspection of morphological characters revealed that many of the species split to multiple BINs as a result of their high intraspecific sequence divergence showed morphological differences between individuals in different clusters, suggesting that they represent more than one species.

## Discussion

This study has tested the effectiveness of DNA barcodes as a tool for the identification of a substantial fraction of the coleopteran fauna of northern Europe. Two of the four extant suborders of beetles are missing from our data as the Archostemata are not found in North Europe and the Myxophaga are only represented by a single very rare species (*Sphaerius acaroides* Waltl, 1838) [29]. However, all major lineages in the other suborders (Adephaga, Polyphaga), such as Carabidae and Dytiscidae, as well as the megadiverse Staphylinidae and Chrysomeloidea, are well represented. The proportion of species with diagnostic barcode sequences was very high, 98.3%. The few cases of barcode identification failures we observed all involve closely related species that are often difficult to identify by morphological characters as well. In fact, even the species status of some of these taxa remains controversial. For example, the staphylinid *Rybaxis laminata* (Motschulsky, 1836) was considered synonymous to *R. longicornis* (Leach, 1817) by Besuchet [37], but was recently separated based on differences in male genitalia as well as external male characters, although the females are apparently indistinguishable morphologically [38]. Another ambiguous case is the *Cakile*-feeding monophagous weevil *Ceutorhynchus cakilis* (Hansen, 1917) which is distinguished from its widespread, common and polyphagous sister species *C. typhae* (Herbst, 1795) only by its slightly larger size and a small difference in elytral pubescence [39]. However, oversplitting of species seems to account for only a minority of the 15 cases of barcode sharing by congeners that we observed. Most of these taxa likely reflect incomplete lineage sorting after recent speciation. Another possible explanation for haplotype sharing is introgression, but distinguishing between the two would require more detailed genetic analysis including nuclear markers.

Although no prior study has examined such a large number of beetle species, the analysis of Central European Carabidae found a similar identification success (73 of 75 species readily identifiable by DNA barcodes) [19]. Success rates of 100% have been reported for some animal groups, but these have generally dealt with many fewer species (7–260 species of birds and various insect groups studied by [40], [41], [42], [43] and [20]). The Lepidoptera of Eastern North America were studied on a similar scale by Hebert *et al*. [44], with 99.3% of 1327 sampled species possessing diagnostic DNA barcodes. A recent study on Noctuoidea [45] found that 90% of 1541 species (representing 99.1% of the Canadian noctuoid moth species) could be unambiguously identified on a national scale, and 95.6% on a provincial scale.

The nearest-neighbor divergences between the species in our study are considerably higher than those detected in other groups. This difference may be partially due to sampling issues discussed below. However, when compared with broad-scale studies on other animal groups such as birds [20] and Lepidoptera [44], the divergences in Coleoptera are markedly higher. The comparison of between-species divergences is slightly complicated because early barcoding studies often reported mean instead of minimum divergences between species, overestimating the barcoding gap [46]. The current barcode gap analysis tool in BOLD uses nearest neighbor distances. The overall average K2P divergence between nearest neighbor species was 11.99% in our data, and generally higher in lineages of Polyphaga (Table S1). This is greater than the value reported by Hebert *et al*. [44] as average divergence between species for most Lepidopteran families (mean between-species divergence over all families was 11.72% in that study). Zahiri et al. [45] report average K2P nearest neighbor divergences ranging from 3.01% to 5.80% within families of Canadian Noctuoidea. At least between these two major insect orders, the difference in divergences seems to be real with NN distances much higher in Coleoptera than in Lepidoptera. This pattern may reflect differences in the average age of species, different mutation rates for COI in Coleoptera and Lepidoptera, or differences in nucleotide composition and patterns of nucleotide substitution.

Because allopatric speciation appears to be dominant in the animal kingdom, sister species often show little or no range overlap [47]. As a result, any regional study, such as ours, will often fail to include the sister taxa for many of the species analyzed. As the geographical scale of sampling increases, the maximum intraspecific divergence should increase and the distance to the nearest neighbor should decrease as more closely related species pairs are sampled and more of the total variation within species is observed. Bergsten *et al.* [24] observed this pattern in their work on Dytiscidae in the western Palearctic region, and Hausmann *et al.* noted a similar trend in a Europe-wide survey of geometrid moth barcodes [25]. The proportion of non-monophyletic species also increased along with the geographic range of sampling in both studies. In geographically restricted subsets, both Hausmann *et al.* [25] and Bergsten *et al*. [24] observed high success of barcode identification. However, expansion of geographical scale also creates problems in drawing boundaries between species and hence the interpretation of results. The species status of allopatric populations is difficult to assess as the biological species concept can only be applied to populations in sympatry. This problem was discussed by Mutanen *et al*. [48] who showed that several Arctic-Alpine species pairs of Lepidoptera have apparently been incorrectly considered as different species, while cases of cryptic diversity and misidentifications impeded data interpretation in other cases. Bergsten *et al.* [24] suggested that the incorporation of species’ geographical range data into the global barcode database would improve the accuracy of identification queries, but also note that the geographical restriction of identification queries would be problematic in some situations such as quarantine programs aimed at the interception of closely allied species.

The effects of sampling on observed genetic divergences both within and among species are clearly visible even on a local scale. Incomplete sampling of species within a clade will inevitably lead to overestimation of the barcode gap. Our resampling study on the Carabidae indicated that nearest neighbor distances dropped by about 30% (10.7% to 7.5%) as species coverage rose 9-fold (from 20 to 180 species). Our work also provides tentative evidence of differences in nearest neighbor (NN) distances between families in the two major suborders of Coleoptera as evidenced by average NN distances of 7.28% in Carabidae and 8.66% in Dytiscidae, major families of Adephaga, versus 11.37% in Staphylinidae and 11.74% in Chrysomelidae, two families of Polyphaga. Interestingly, prior studies have suggested that the rate of nucleotide substitution in mitochondrial genes is lower in the suborder Adephaga than in Polyphaga [49]. This pattern further implies that the effects of geographical scale on barcode differentiation might be different between Adephaga and Polyphaga.

Our studies indicated that the maximum divergence within a species increased in a slow but steady fashion with the number of specimens analyzed. Bergsten *et al.* [24] showed that observing 95% of the genetic variation within the diving beetle species *Agabus bipustulatus* (Linnaeus, 1767) on a continental scale required approximately 250 specimens if sampling was random, and 70 specimens if the sampling strategy was optimized to examine geographical variation. Required sample sizes are certainly much smaller for regional scale studies such as ours, but we undoubtedly only observed a fraction of the total regional genetic variation within most species. However, because NN divergences between species are generally high, our estimates of barcode identification success are unlikely to be substantially reduced, even with increased regional sampling.

Several barcoding studies ([25], [50], [45]) have found previously overlooked species even in taxa and regions presumed to be thoroughly studied. This was also the case in the present study as 5.7% of the species formed two or more distinct barcode clusters, some of which were found to clearly differ in their morphology as well. For example, our specimens of *Hydrobius fuscipes* (Linnaeus, 1758) (Hydrophilidae) were divided into three BINs that upon closer inspection perfectly corresponded with the three “varieties” of this species known from Northern Europe (*subrotundus* Stephens, 1829, *rottenbergii* Gerhardt, 1872 and *fuscipes* s.str.). Fossen [51] has recently studied this species complex more thoroughly, combining morphological characters with one mitochondrial and two nuclear loci and concluded that the three “varieties” are indeed different species. *Dictyoptera aurora* (Herbst, 1784) (Lycidae) is divided into two BINs with differences in pronotal shape, while *Cassida nobilis* Linnaeus, 1758 (Chrysomelidae) includes two BINs whose component specimens possess distinctly different body outlines and coloration. These and other similar cases will be examined in more detail in later publications with broader geographic sampling, more barcoded specimens and detailed morphological study. Besides overlooked species, some deep intraspecific splits may reflect infections by multiple strains of *Wolbachia* or other microbial endosymbionts [52] or the admixture of populations that were formerly isolated in different glacial refugia.

The North European beetle fauna is relatively small and among the most thoroughly studied in the world, with a long history of intensive taxonomic research dating back to Linnaeus. Despite this work, our studies have revealed the likely occurrence of at least 20 species overlooked by current taxonomy. More importantly, nearly all (98.3%) of the sampled species can be reliably identified with DNA barcodes. The larvae of many beetle species are difficult to identify or completely undescribed which has served as a barrier to the construction of detailed food webs. As shown by Wirta *et al.* [53], DNA barcoding can complement or even surpass traditional methods of food web construction even when the species involved are few and readily identifiable by morphology. Beetles are often targeted in forest ecology studies as beetles, trees and fungi form a well-known “ecological triangle” [5]. The identification of specimens currently consumes considerable time and money, results that could be obtained more efficiently by coupling next generation sequencing techniques with a comprehensive DNA barcode library, similar to the approach employed for biomonitoring of aquatic insects by Hajibabaei *et al.* [54].

DNA barcodes show very high potential as an identification tool for Coleoptera, and also as an addition to the beetle taxonomist’s toolkit. Even in the few cases where barcodes fail to deliver a species identification in this study, it narrows the options to a pair (or trio in one case) of closely related species. Especially for larval identification, this is a very encouraging result. Mapping the diversity and life histories of Coleoptera is a key to deeper understanding of ecosystem structure and function. The present results should encourage further beetle barcoding projects at various geographical and taxonomic scales. As DNA barcodes appear to distinguish beetle species very efficiently, our results give hope for a global identification system for this group. Certainly, the mapping of undescribed beetle diversity will be greatly expedited by making DNA barcoding a routine part of the process.

## Supporting Information

Table S1 **List of species names, countries of origin, BOLD sample IDs, and GenBank accession numbers of the analyzed specimens.**
(XLSX)

Table S2 **Family-wise summary of divergence within species, nearest neighbor distances and numbers of species and individuals analyzed.**
(XLSX)
